# Electrochemical immunosensor with Cu(I)/Cu(II)-chitosan-graphene nanocomposite-based signal amplification for the detection of newcastle disease virus

**DOI:** 10.1038/s41598-020-70877-3

**Published:** 2020-08-17

**Authors:** Jiaoling Huang, Zhixun Xie, Yihong Huang, Liji Xie, Sisi Luo, Qing Fan, Tingting Zeng, Yanfang Zhang, Sheng Wang, Minxiu Zhang, Zhiqin Xie, Xianwen Deng

**Affiliations:** 1 Guangxi Key Laboratory of Veterinary Biotechnology, Guangxi Veterinary Research Institute, 51 You Ai North Road, Nanning, 530001 Guangxi China; 2 Liuzhou Centre for Animal Disease Control and Prevention, Beijing, China

**Keywords:** Analytical chemistry, Immunochemistry, Graphene

## Abstract

An electrochemical immunoassay for the ultrasensitive detection of Newcastle disease virus (NDV) was developed using graphene and chitosan-conjugated Cu(I)/Cu(II) (Cu(I)/Cu(II)-Chi-Gra) for signal amplification. Graphene (Gra) was used for both the conjugation of an anti-Newcastle disease virus monoclonal antibody (MAb/NDV) and the immobilization of anti-Newcastle disease virus polyclonal antibodies (PAb/NDV). Cu(I)/Cu(II) was selected as an electroactive probe, immobilized on a chitosan-graphene (Chi-Gra) hybrid material, and detected by differential pulse voltammetry (DPV) after a sandwich-type immune response. Because Gra had a large surface area, many antibodies were loaded onto the electrochemical immunosensor to effectively increase the electrical signal. Additionally, the introduction of Gra significantly increased the loading amount of electroactive probes (Cu(I)/Cu(II)), and the electrical signal was further amplified. Cu(I)/Cu(II) and Cu(I)/Cu(II)-Chi-Gra were compared in detail to characterize the signal amplification ability of this platform. The results showed that this immunosensor exhibited excellent analytical performance in the detection of NDV in the concentration range of 10^0.13^ to 10^5.13^ EID_50_/0.1 mL, and it had a detection limit of 10^0.68^ EID_50_/0.1 mL, which was calculated based on a signal-to-noise (S/N) ratio of 3. The resulting immunosensor also exhibited high sensitivity, good reproducibility and acceptable stability.

## Introduction

Newcastle disease virus (NDV) is a viral disease of poultry that belongs to avian paramyxovirus 1. It is a single-strand, non-segmented, and negative-sense RNA virus^1^, and it is a great threat to the poultry industry^2^. The first important step in NDV prevention and control is to develop a rapid and sensitive method for diagnosis. Currently, several methods for detecting NDV, included virus isolation^3^, reverse transcription polymerase chain reaction (RT-PCR)^4^, real-time RT-PCR^5^, immunochromatographic strip (ICS) tests^6^, and reverse transcription loop-mediated isothermal amplification (RT-LAMP) assays^7^, have been reported. However, these diagnostic methods had some disadvantages; for example, virus isolation is the gold standard for the detection of NDV, but the procedure is time-consuming. For RT-PCR, appropriate laboratory facilities and a trained technician are needed. Real-time RT-PCR requires complicated operations as well as expensive reagents and equipment. Therefore, these diagnostic methods are limited in practical applications.

Electrochemical immunosensors are powerful tools that have good specificity, high sensitivity, good precision, and simple instrumentation; give rapid and reliable responses; and are relatively low cost. Their use in clinical diagnosis, food analysis, environmental monitoring and archaeological studies should be highly valuable^8^. Furthermore, electrochemical immunosensors are based on antibody-antigen reactions. Therefore, immobilizing antibodies or antigens on a transducer as a biorecognition element plays a very important role in the construction of electrochemical immunosensors. Different methods for immobilizing antibodies/antigens on a transducer, including chemical and physical adsorption, have been discussed^9^. It has been reported that chitosan (Chi) is a suitable matrix for immobilizing biorecognition elements due to its biocompatibility, hydrophilicity, mouldability, chemical reactivity, and biodegradability^10^. However, Chi is non-conductive and has low solubility in different solutions; thus, many kinds of nanomaterials have been combined with Chi to increase its conductivity for the fabrication of electrochemical immunosensors^11^. Modifying transducers with conductive materials enhances the electron transfer between the electrode surface and electrolyte^10,12,13^. Furthermore, modifying them with nanomaterials provides a rougher surface that enables the biorecognition element to attach closely to the electrode surface. Many kinds of nanomaterials, including Gra^14^, multi-walled carbon nanotubes^15^, gold nanoparticles^12^, magnetic nanoparticles^16^, quantum dots^17^ and hybrid nanostructures^18^, have been used in immunosensors.

Gra has a one-atom-thick planar structure composed of sp^2−^ hybridized carbon atoms packed in a honeycomb-like lattice^19^. Due to this unique structure, Gra has an exceptionally high surface-to-volume ratio, electrical conductivity, and thermal conductivity and good mechanical properties^20^. Gra has been used to improve the sensitivity and stability of immunosensors many times^21,22^. However, the direct immobilization of protein molecules on Gra is difficult. As previously mentioned, Chi can easily immobilize protein molecules and form a film on transducers. Due to these properties, nanocomposites consisting of Chi and Gra are an ideal immunosensor material, and our group successfully synthesized a silver nanoparticle-chitosan-graphene composite to construct an electrochemical immunosensor^23^.

However, copper is much less expensive than silver nanoparticles, and Cu(II) ions can be adsorbed by Chi from aqueous solutions via chelation because of its unique three-dimensional structure^24^. Additionally, the synthesis of CuO (Cu(II)) and Cu_2_O (Cu(I)) using Chi as a stabilizing and reducing agent has been reported^25–27^. Furthermore, Cu(II) ions provide a good stripping voltammetric signal^28^. In addition, Cu(I) has a direct band gap of 2.0 eV and is a p-type semiconductor that is very important in superconductors and electrode materials^26,27^. As previously mentioned, Cu(I) and Cu(II) can be used as electroactive materials. The more electroactive a material carried by an immunosensor is, the more sensitive the immunoassay is. Therefore, in this study, Gra, which has a high loading capacity, was used to load a large amount of electroactive probes on an immunosensor. Hybrid Cu(I)/ Cu(II)-modified Gra effectively amplifies signals. In this work, a sandwich-type electrochemical immunosensor was designed using a gold nanoparticle-chitosan-graphene (AuNP-Chi-Gra) nanocomposite as the platform and a Cu(I)/Cu(II)-chitosan-graphene (Cu(I)/Cu(II)-Chi-Gra) nanocomposite as the label for detecting NDV with a low detection limit (10^0.68^ EID_50_/0.1 mL) and high sensitivity in a relatively wide linear range (from 10^0.13^ to 10^5.13^ EID_50_/0.1 mL). The developed immunosensor shows potential for applications in the clinical screening of other pathogenic microorganisms and point-of-care diagnostics.

## Results and discussion

### Morphological characterization of the nanocomposites

**Figure 1:**
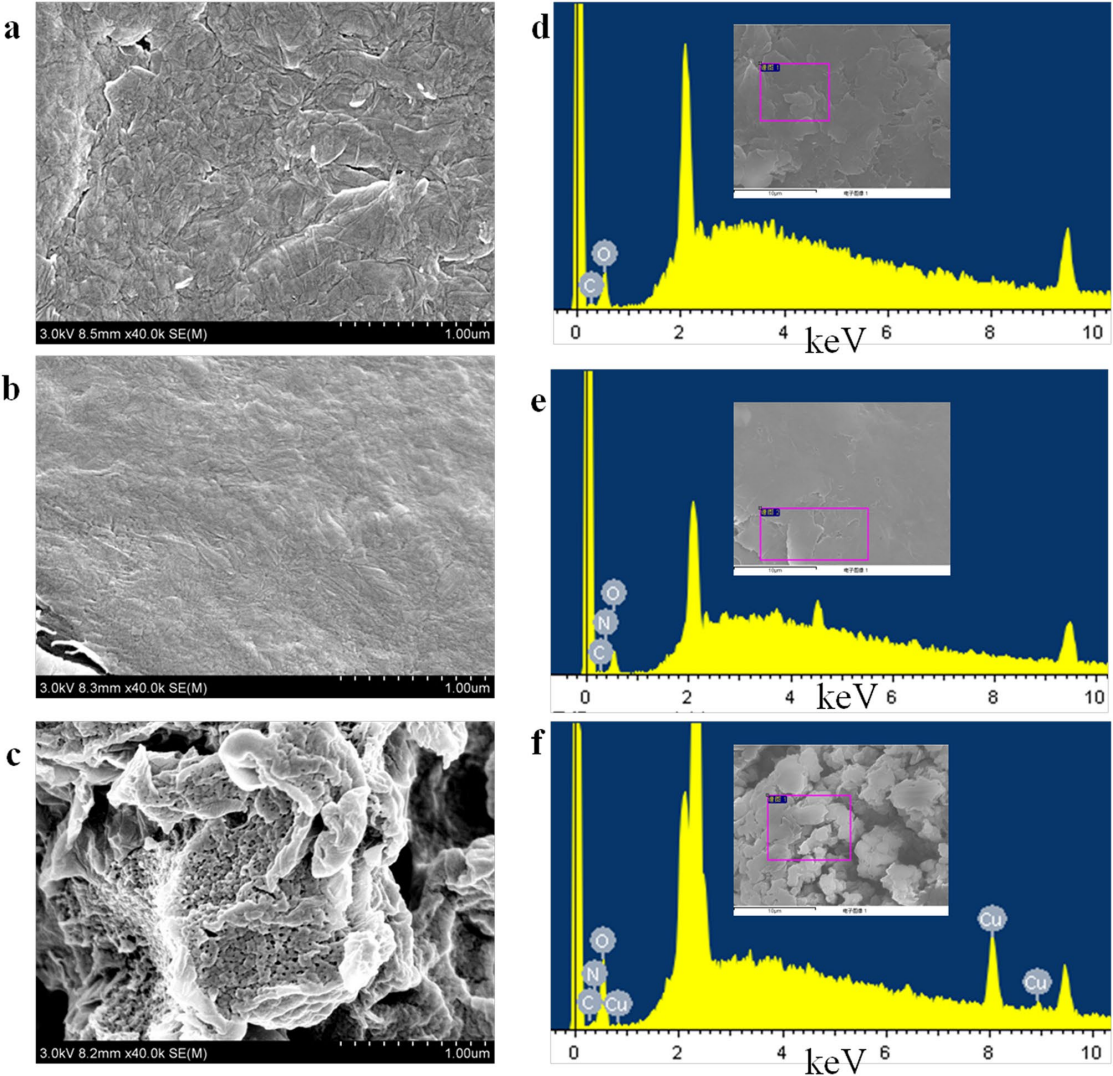
SEM images of Gra (**a**), Chi-Gra (**b**) and Cu(I)/Cu(II)-Chi-Gra (**c**). The zones examined by EDS (marked by red boxes) and the results of the analysis are also shown for the Gra (**d**), Chi-Gra (**e**) and Cu(I)/Cu(II)-Chi-Gra (**f**) samples.

Figure 1 shows scanning electron microscopy (SEM) images and energy dispersive spectrometry (EDS) analyses of Gra, Chi-Gra and Cu(I)/Cu(II)-Chi-Gra. The image of Gra confirms that its structure had many folds (a). After Gra was modified with Chi, the folded structure was filled with Chi, and the surface of the Chi-Gra composite became smooth (b). The presence of Chi on Gra was confirmed by EDS analysis (e). N was observed in the sample because Chi is a natural, biocompatible polymer with many amino groups. Interestingly, the Cu(I)/Cu(II)-Chi-Gra nanocomposite exhibited many upturned folded edges and had a porous matrix (c). Due to this characteristic structure, the exposed surface of the Cu(I)/Cu(II)-Chi-Gra nanocomposite was larger than those of the Chi-Gra composite and Gra. The active surface area increased, resulting in a high surface/volume ratio for antibody immobilization. Furthermore, this porous structure facilitated electrochemical signal amplification. The successful incorporation of Cu(I)/Cu(II) into the Chi-Gra surface was also confirmed by EDS analysis (f).

### Chemical characterization of the nanocomposites

**Figure 2:**
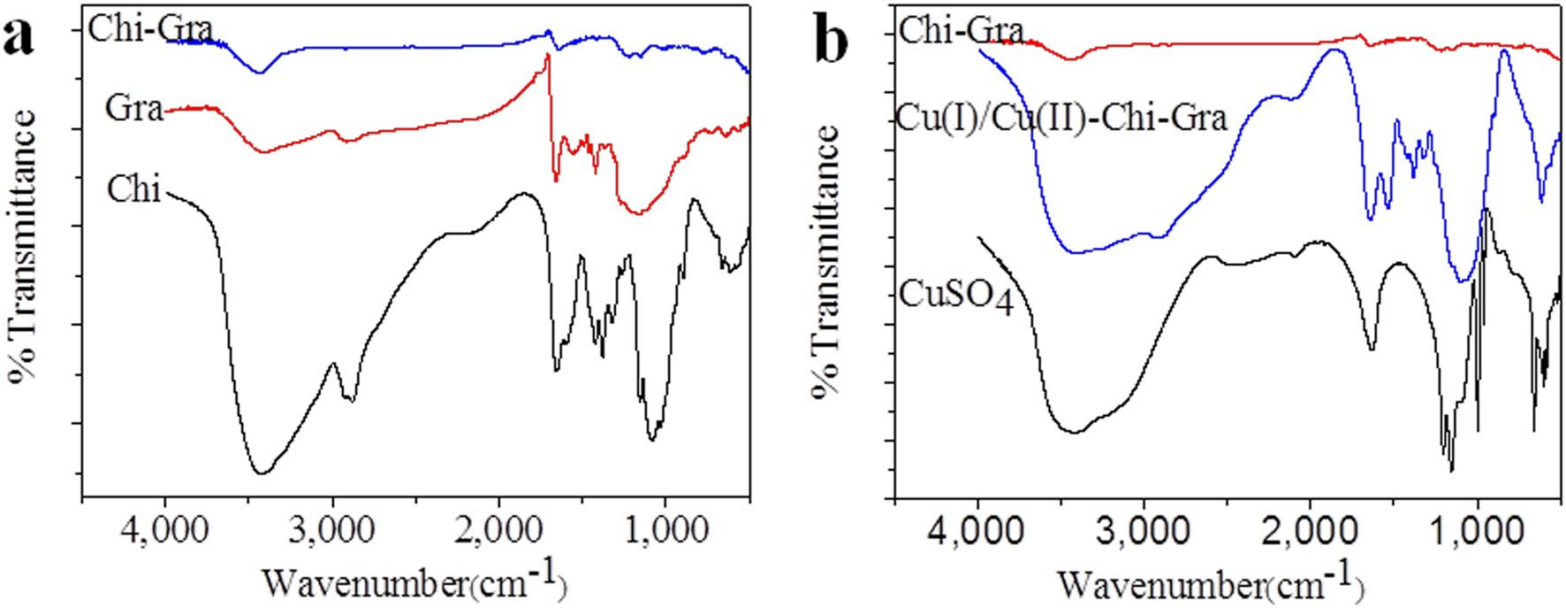
FT-IR spectra of (**a**) Chi, Gra, and Chi-Gra and (**b**) Chi-Gra, CuSO_4_, and Cu(I)/Cu(II)-Chi-Gra.

Fourier transform infrared (FT-IR) spectra of Chi, Gra, Chi-Gra, CuSO_4_ and Cu(I)/Cu(II)-Chi-Gra are presented in Fig. 2. As shown in Fig. 2a (black line, Chi), the stretching vibrations of the –OH bonds in Chi were observed at 3,425 cm^−1^, and this band overlapped with the –NH_2_ stretching peaks^29^. The signals originating from the C-H stretching vibrations were observed at approximately 2,920 cm^−1^ and 2,878 cm^−1^^30^. The NH_2_ group and γ-NH_2_ bending vibrations appeared at 1653 cm^−1^ and 1596 cm^−1^, respectively^31^. Furthermore, the peak at 1,424 cm^−1^ was attributed to the OH bending vibration. The stretching vibrations of the C–C–O bonds in the Chi backbone were observed at approximately 1,154 cm^−1^, 1,081 cm^−1^ and 1,034 cm^−1^. As shown in Fig. 2a (red line), the characteristic absorption bands of pure Gra appeared at 1555 cm^−1^, 1,459 cm^−1^, and 1,420 cm^−1^ (benzene ring backbone stretching vibrations); 1659 cm^−1^ (C=O stretching vibration); 2,916 cm^−1^ (C–H stretching vibration); and 3,406 cm^−1^ (O–H stretching vibration). Chi adsorption on Gra resulted in the appearance of the characteristic absorption bands of pure Gra in the FT-IR spectrum of Chi-Gra (Fig. 2a; blue line), but compared with pure Gra, the characteristic absorption bands of Chi-Gra had lower intensities, which helped confirm that Chi was successfully adsorbed on Gra. Comparing the spectra of Chi-Gra and Cu(I)/Cu(II)-Chi-Gra (Fig. 2b; red and blue lines) revealed some changes in the intensities and shifts in the peaks. Furthermore, the main absorption peaks of pure CuSO_4_ (Fig. 2b; black line) were also observed in the FT-IR spectrum of Cu(I)/Cu(II)-Chi-Gra (Fig. 2b; blue line), providing evidence of the interaction between CuSO_4_ and Chi-Gra. Chi-Gra binds Cu^2+^ well because Chi-Gra contains many negatively charged groups (carboxylic (O=C–OH), hydroxyl (–C–OH) and carbonyl (–C=O)) that can strongly interact with the positively charged Cu^2+^ ion in CuSO_4_.

**Figure 3:**
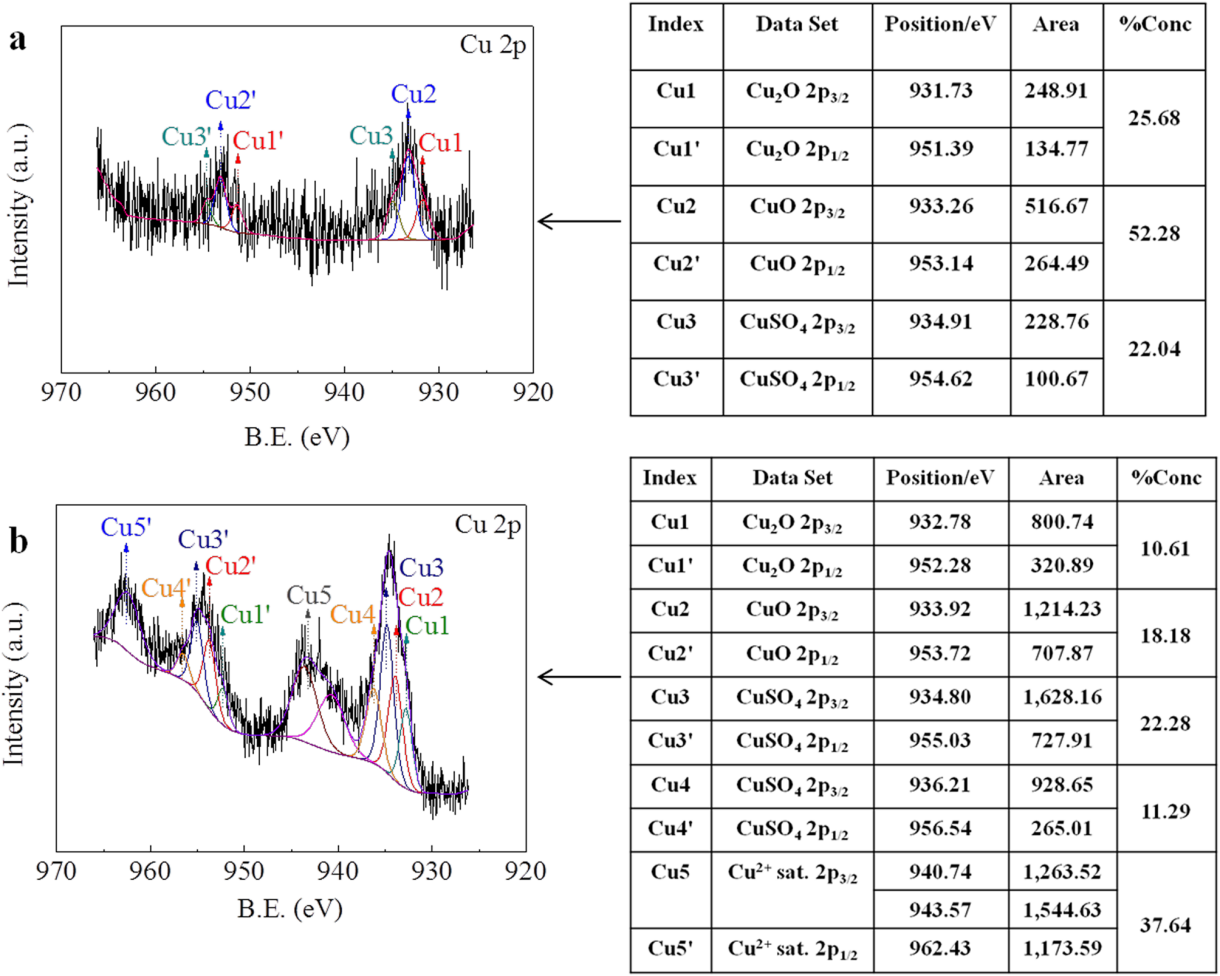
XPS spectra of (**a**) Cu(I)/Cu(II)-Chi-Gra and (**b**) rich[Cu(I)/Cu(II)]-Chi-Gra.

In addition, X-ray photoelectron spectroscopy (XPS) was used to identify the valence state of Cu. The XPS spectrum of Cu(I)/Cu(II)-Chi-Gra is shown in Fig. 3a. The formation of Cu_2_O was confirmed by the presence of the Cu 2p_3/2_ peak at 931.73 eV and the Cu 2p_1/2_ peak at 951.39 eV^32^. Furthermore, the presence of Cu 2p_3/2_ and Cu 2p_1/2_ peaks with binding energies of 933.26 eV and 953.14 eV, respectively, proved the formation of CuO^32^. The presence of CuSO_4_ was confirmed by the Cu 2p_3/2_ peak at 934.91 eV and Cu 2p_1/2_ peak at 954.62 eV^33^. In addition, to obtain a clearer XPS survey, 10 times the amount of CuSO_4_ was added to Chi-Gra to prepare rich[Cu(I)/Cu(II)]-Chi-Gra, and the XPS spectrum of rich[Cu(I)/Cu(II)]-Chi-Gra shown in Fig. 3b confirmed that the valence states of the Cu element were Cu^+^ (Cu(I)) and Cu^2+^ (Cu(II)). The concentration of Cu(II) in rich[Cu(I)/Cu(II)]-Chi-Gra was higher than that in Cu(I)/Cu(II)-Chi-Gra because the ability of Chi to chelate Cu^2+^ is stronger than the ability of Chi to reduce Cu^2+^ to Cu^+^. Additionally, the presence of Cu4, Cu4′, Cu5 and Cu5′ in rich[Cu(I)/Cu(II)]-Chi-Gra might be due to the different Cu^2+^-chelating abilities of the various functional groups in Chi-Gra. Under competitive conditions, functional groups with a stronger Cu^2+^-chelating ability chelate Cu^2+^ first, and functional groups with a weaker Cu^2+^-chelating ability chelate Cu^2+^ last. When the amount of Cu^2+^ is too low, the functional groups with a weaker Cu^2+^-chelating ability lose Cu^2+^, but these functional groups can chelate Cu^2+^ when a sufficient amount of Cu^2+^ is present. Therefore, Cu4, Cu4′, Cu5 and Cu5′ were present in rich[Cu(I)/Cu(II)]-Chi-Gra, but absent in Cu(I)/Cu(II)-Chi-Gra.

### Electrochemical characterization of the immunosensor

**Figure 4:**
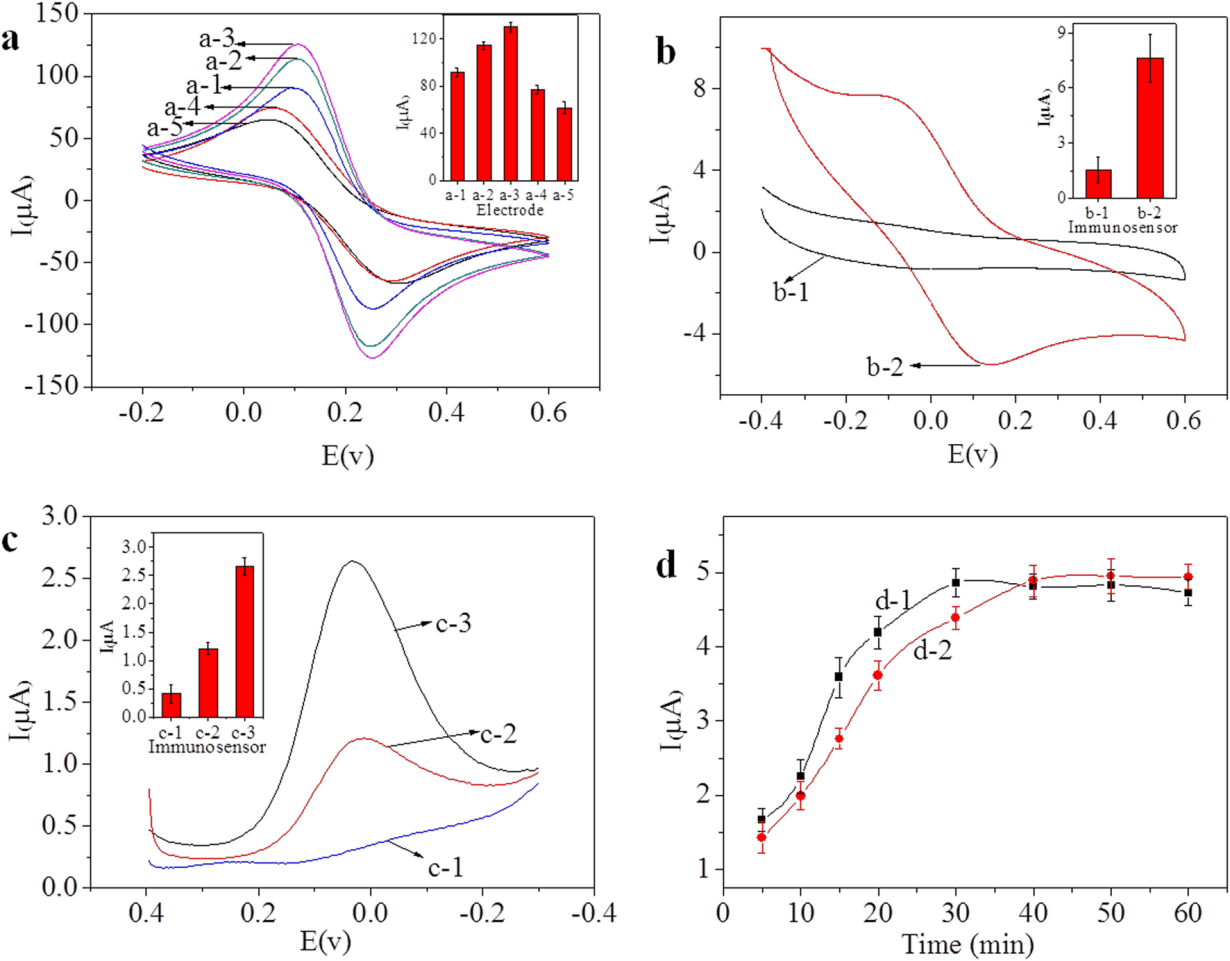
(**a**) CV curves of the electrode at different stages obtained at a scan rate of 50 mV/s: (a-1) GCE, (a-2) AuNP-Chi-GCE, (a-3) AuNP-Chi-Gra-GCE, (a-4) MAb/NDV-AuNP-Chi-Gra-GCE, and (a-5) BSA-MAb/NDV-AuNP-Chi-Gra-GCE. The supporting electrolyte was 5 mM Fe(CN)_6_^3−/4−^ + 0.1 M KCl + 0.01 M PBS (pH = 7.4). (**b**) CV curves of the immunosensor measurement process: (b-1) BSA-MAb/NDV-AuNP-Chi-Gra-GCE and (b-2) PAb/NDV-Cu(I)/Cu(II)-Chi-Gra-NDV-BSA-MAb/NDV-AuNP-Chi-Gra-GCE. The supporting electrolyte was 0.1 M KCl + 0.01 M PBS (pH = 7.4). The sample included 15 µL of 10^5.13^ EID_50_/0.1 mL NDV (F48E9). (**c**) DPV of the immunosensor measurement process: (c-1) BSA-MAb/NDV-AuNP-Chi-Gra-GCE, (c-2) PAb/NDV-Cu(I)/Cu(II)-Chi-NDV-BSA-MAb/NDV-AuNP-Chi-Gra-GCE, and (c-3) PAb/NDV-Cu(I)/Cu(II)-Chi-Gra-NDV-BSA-MAb/NDV-AuNP-Chi-Gra-GCE. The supporting electrolyte was 0.1 M KCl + 0.01 M PBS (pH = 7.4). The sample included 15 µL of 10^2.13^ EID_50_/0.1 mL NDV (F48E9). (**d**) Influence of the incubation time on the current response of the immunosensor to (d-1) NDV and (d-2) PAb/NDV-Cu(I)/Cu(II)-Chi-Gra. The sample included 15 µL of 10^5.13^ EID_50_/0.1 mL NDV (F48E9).

Cyclic voltammetry (CV) was used to investigate the surface of the glassy carbon electrode (GCE) during the process. The electrochemical behaviour was monitored in 5 mM Fe(CN)_6_^3−/4−^ (1:1) and 0.01 M phosphate-buffered saline (PBS) (pH = 7.4, containing 0.1 M KCl) in the potential range of − 0.2 to 0.6 V at a scan rate of 50 mV/s^−1^, and the results are shown in Fig. 4a. A pair of well-defined voltammetric peaks was obtained for the bare GCE (curve a-1). Coating the bare GCE with AuNP-Chi (curve a-2) and AuNP-Chi-Gra (curve a-3) caused an increase in the redox peak current. A comparison of the curves indicated that the AuNPs and Gra had good conductivity and electrocatalytic effects. After attaching MAb/NDV to the modified GCE (curve a-4), the current decreased. This decrease can be explained by the following two factors: (i) AuNP-Chi-Gra could conjugate MAb/NDV via Au–S covalent bonds, and (ii) electron transfer was hindered by MAb/NDV. Subsequently, BSA was used to block the immunosensor, and the redox peaks decreased even further (curve a-5), because BSA is hydrophobic and electron transfer was further inhibited.

To investigate the immunosensor detection programme, CV was performed in 0.01 mmol/L PBS (pH = 7.4) containing 0.1 mmol/L KCl, and the results are shown in Fig. 4b. For CV curve b-1 in Fig. 4b, which was obtained with the BSA-MAb/NDV-AuNP-Chi-Gra film-modified GCE, the background current was low, and no CV redox waves were observed because of the absence of electrochemically active substances in the working solution. After the immunosensor was incubated with 10^5.13^ EID_50_/0.1 mL NDV and sandwiched for the immunoreaction with PAb/NDV-Cu(I)/Cu(II)-Chi-Gra, stable redox peaks were observed at 0.13 and − 0.08 V vs. saturated calomel electrode (SCE) (curve b-2 in Fig. 4b), due to the redox reaction of Cu(I)/Cu(II). The peak at 0.13 V was caused by the oxidation of Cu(I) to Cu(II), and the reduction of Cu(II) to Cu(I) produced the peak at − 0.08 V. These results indicated the efficient redox activity of Cu(I)/Cu(II)-functionalized Gra.

### Comparison of different signal amplification strategies

Signal amplification strategies are very important for immunosensors. Two signal label materials (PAb/NDV-Cu(I)/Cu(II)-Chi-Gra and PAb/NDV-Cu(I)/Cu(II)-Chi) were prepared, and differential pulse voltammetry (DPV) was performed from − 0.3 to 0.4 V at a 50 mV/s scan rate using a 10^2.13^ EID_50_/0.1 mL sample to evaluate the effects of the signal amplification materials. The results are shown in Fig. 4c. As indicated by curve c-1, in the absence of a signal labelling material, a low background current was obtained, and no anodic peak was observed for the immunosensor. In contrast, the immunosensor conjugated with PAb/NDV-Cu(I)/Cu(II)-Chi-Gra (curve c-3) exhibited a greater current shift than the immunosensor conjugated with PAb/NDV-Cu(I)/Cu(II)-Chi (curve c-2). The increase in the current shift was due to the use of Gra, which has with a high surface/volume ratio, as the carrier, leading to the immobilization of Cu(I)/Cu(II) on the GCE and facilitating electrochemical signal amplification. These results confirmed that the immunosensor with Gra could load more of the electroactive signal labelling material and PAb/NDV than the immunosensor without Gra. Accordingly, the signal of the immunosensor was greatly amplified by using Gra.

### Optimization of the experimental conditions

During NDV capture and the specific reaction with the signal labelling material (PAb/NDV-Cu(I)/Cu(II)-Chi-Gra), the incubation time is an important factor. Thus, the incubation times of NDV and PAb/NDV-Cu(I)/Cu(II)-Chi-Gra were optimized separately. To optimize the NDV incubation time, different incubation times (5, 10, 15, 20, 30, 40, 50, and 60 min) were used, and after incubation with NDV, the immunosensors were incubated with PAb/NDV-Cu(I)/Cu(II)-Chi-Gra for 60 min. Finally, the immunosensors were used for DPV detection. Each test was repeated five times. The results are shown in Fig. 4d, curve d-1. As the NDV incubation time was increased up to 30 min, the electrochemical response increased; after 30 min, a constant value was reached, indicating that the immunoreaction was complete, and all the NDV in the sample was captured by the immunosensor. Thus, the optimal incubation time for NDV was 30 min.

To optimize the PAb/NDV-Cu(I)/Cu(II)-Chi-Gra incubation time, the immunosensors were first incubated with NDV (10^5.13^ EID_50_/0.1 mL) for 30 min and then incubated with PAb/NDV-Cu(I)/Cu(II)-Chi-Gra for 5, 10, 15, 20, 30, 40, 50, and 60 min, respectively. Finally, the immunosensors were used for DPV detection. Each test was repeated five times. The results are shown in Fig. 4d, curve d-2. In the second immunoreaction step, as the PAb/NDV-Cu(I)/Cu(II)-Chi-Gra incubation time was increased, the electrochemical response current increased, reaching a steady-state value at 40 min, which indicates that the reaction between NDV and PAb/NDV-Cu(I)/Cu(II)-Chi-Gra was complete. Thus, the optimal incubation time for PAb/NDV-Cu(I)/Cu(II)-Chi-Gra was 40 min. Compared with NDV, PAb/NDV-Cu(I)/Cu(II)-Chi-Gra required more time to complete the reaction, which might be due to the greater steric hindrance of PAb/NDV-Cu(I)/Cu(II)-Chi-Gra.

### Analytical performance of the immunosensor

**Figure 5:**
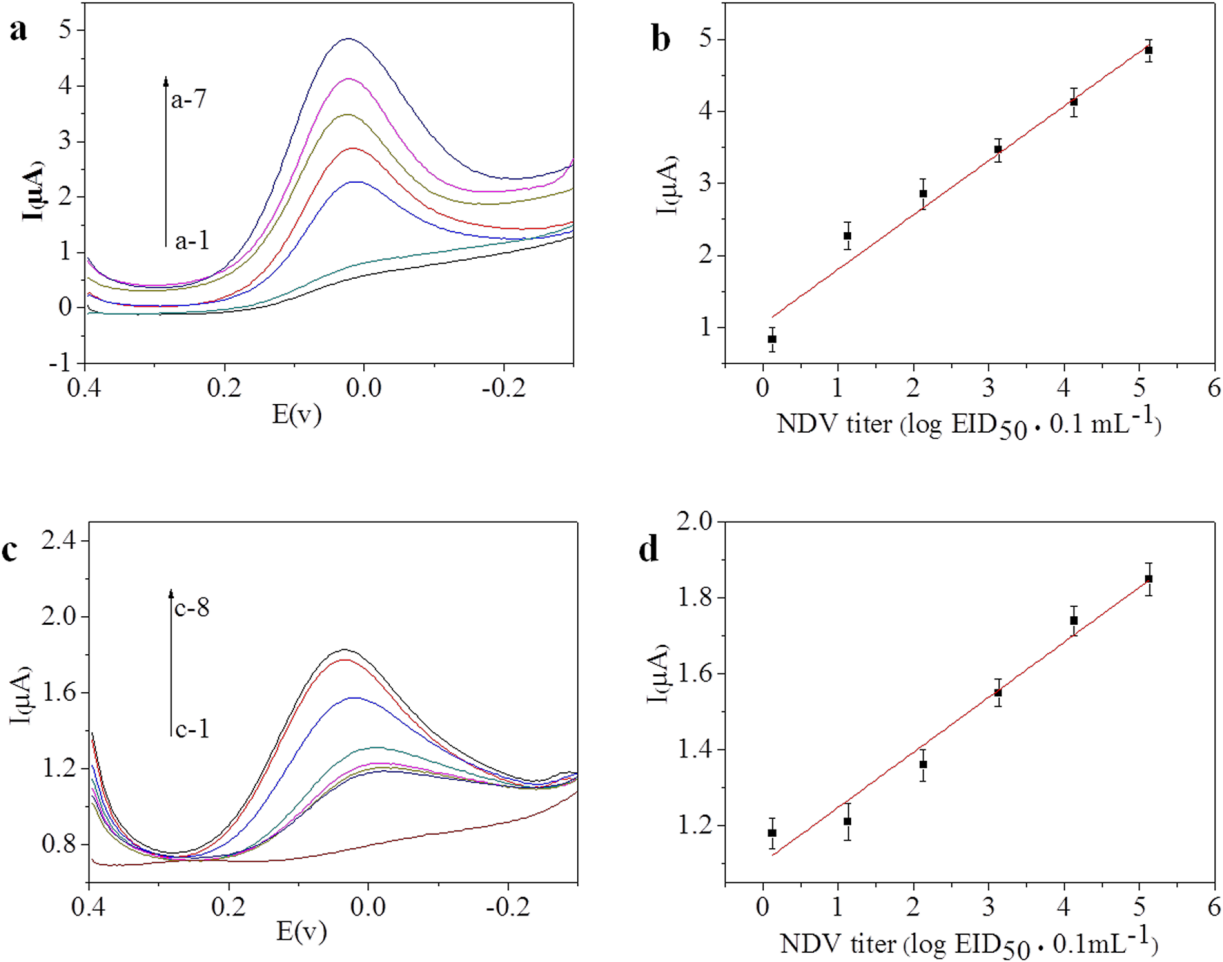
(**a**) Typical DPV signals acquired in the presence of different concentrations of NDV with PAb/NDV-Cu(I)/Cu(II)-Chi-Gra as the label: (a-1) 0, (a-2) 10^0.13^ EID_50_/0.1 mL, (a-3) 10^1.13^ EID_50_/0.1 mL, (a-4) 10^2.13^ EID_50_/0.1 mL, (a-5) 10^3.13^ EID_50_/0.1 mL, (a-6) 10^4.13^ EID_50_/0.1 mL, and (a-7) 10^5.13^ EID_50_/0.1 mL. (**b**) Relationship between the antigen concentration and sensor current response corresponding to (**a**). (**c**) Typical DPV signals before incubation with NDV (c-1) and in the presence of different concentrations of NDV ((c-2) 0, (c-3) 10^0.13^ EID_50_/0.1 mL, (c-4) 10^1.13^ EID_50_/0.1 mL, (c-5) 10^2.13^ EID_50_/0.1 mL, (c-6) 10^3.13^ EID_50_/0.1 mL, (c-7) 10^4.13^ EID_50_/0.1 mL, and (c-8) 10^5.13^ EID_50_/0.1 mL) with PAb/NDV-Cu(I)/Cu(II)-Chi as the label. (**d**) Relationship between the antigen concentration and sensor current response corresponding to (**c**). Error bar =  ± standard deviation.

The response of the prepared immunosensor was measured at different concentrations of NDV (F48E9) under the optimal experimental conditions. The results are shown in Fig. 5a. The electrochemical response current increased as the concentration of NDV increased, and the peak of the electrochemical response current was proportional to the concentration in the range of 10^0.13^ to 10^5.13^ EID_50_/0.1 mL. The linear regression equation, which is shown in Fig. 5b, was I (μA) = 0.75 log EID_50_/0.1 mL + 1.05, with a correlation coefficient of 0.97075, and the limit of determination for NDV was 10^0.68^ EID_50_/0.1 mL, which was calculated based on a signal-to-noise ratio of 3 (S/N = 3). These results demonstrated that the immunosensor was sensitive enough to quantitatively monitor NDV.

The results for the immunosensor with PAb/NDV-Cu(I)/Cu(II)-Chi-Gra as the signal label were compared with those for the immunosensor with PAb/NDV-Cu(I)/Cu(II)-Chi as the signal label, and the results obtained with PAb/NDV-Cu(I)/Cu(II)-Chi are shown in Fig. 5c. The electrochemical response current increased linearly with increasing NDV concentration, and the calibration curve in the range of 10^0.13^ to 10^5.13^ EID_50_/0.1 mL (Fig. 5d) was: I (μA) = 0.15 log EID_50_/0.1 mL + 1.10. The limit of determination for NDV was 10^2.09^ EID_50_/0.1 mL (S/N = 3). This result indicated that Gra can improve the immunosensor sensitivity. In addition, as shown in Fig. 5c (curve c-2), the background signal was high when PAb/NDV-Cu(I)/Cu(II)-Chi was used as the signal label because without Gra, the excess Chi could not be removed from PAb/NDV-Cu(I)/Cu(II)-Chi by centrifugation, and the excess Chi chelated with Cu(I)/Cu(II) was attached to the GCE by non-specific binding.

### Comparison of methods

The results of a comparative study between the designed method and other methods for NDV detection are summarized in Table 1a. The table shows that the developed electrochemical immunosensor has acceptable sensitivity and advantages over the other methods in terms of rapid detection, intuitiveness, user-friendliness and cost.

**Table 1:** Comparison of the proposed immunosensor with other sensors for NDV detection (a); results of clinical samples (b); analysis data sheet of positive samples (c); recovery results of clinical samples with different concentrations of NDV (d).

(a) Method	Detection time	Detection limit	References		
Virus isolation	4–7 days	1 EID_50_/mL	^3^		
RT-PCR	5 h	10^4.0^ EID_50_/0.1 mL	^4^		
Real-time RT-PCR	3 h	10^1^ EID_50_/mL	^5^		
ICS	15 min	10^4.9^ EID_50_/0.1 mL	^6^		
RT-LAMP	3 h	1.3 Haemagglutination units	^7^		
Proposed immunosensor	70 min	10^0.68^ EID_50_/0.1 mL	This study		

(b) Method	Total number of samples	Number of positive samples	Positive rate/%		
Proposed immunosensor	120	7	5.8		
Virus isolation	120	7	5.8		

(c) NO	Results of the proposed immunosensor			Results of virus isolation	
	Measured concentration/EID_50_/0.1 mL	Average/EID_50_/0.1 mL	RSD/% (n = 5)		
1	40.74, 39.90, 41.27, 39.15, 42.65	40.74	3.28	Positive	
2	92.47, 90.73, 93.04, 91.38, 94.31	92.39	1.52	Positive	
3	107.46, 105.92, 108.17, 110.29, 109.67	108.30	1.61	Positive	
4	367.41, 370.35, 361.91, 374.34, 354.73	365.75	2.09	Positive	
5	409.32, 417.93, 406.78, 423.32, 428.46	417.16	2.19	Positive	
6	742.16, 737.59, 731.81, 749.19, 728.94	737.94	1.10	Positive	
7	1,490.28, 1,481.38, 1,463.57, 1,447.34, 1,452.85	1,467.08	1.25	Positive	

(d) NO	Initial NDV concentration in sample/EID_50_/0.1 mL	Added NDV amount/EID_50_/0.1 mL	Total found		Recovery rate/% (n = 5)
			Average/EID_50_/0.1 mL	RSD/% (n = 5)	
1	40.74	50	87.36	2.74	96.28
2	92.51	100	190.83	2.38	99.13
3	108.30	500	610.17	1.76	100.31
4	365.75	1,000	1,363.72	1.47	99.85
5	417.16	5,000	5,421.03	2.39	100.07
6	737.94	10,000	11,219.82	3.56	104.49
7	1,467.08	50,000	50,734.94	2.71	98.58

### Selectivity, repeatability, reproducibility and stability of the immunosensor

**Figure 6:**
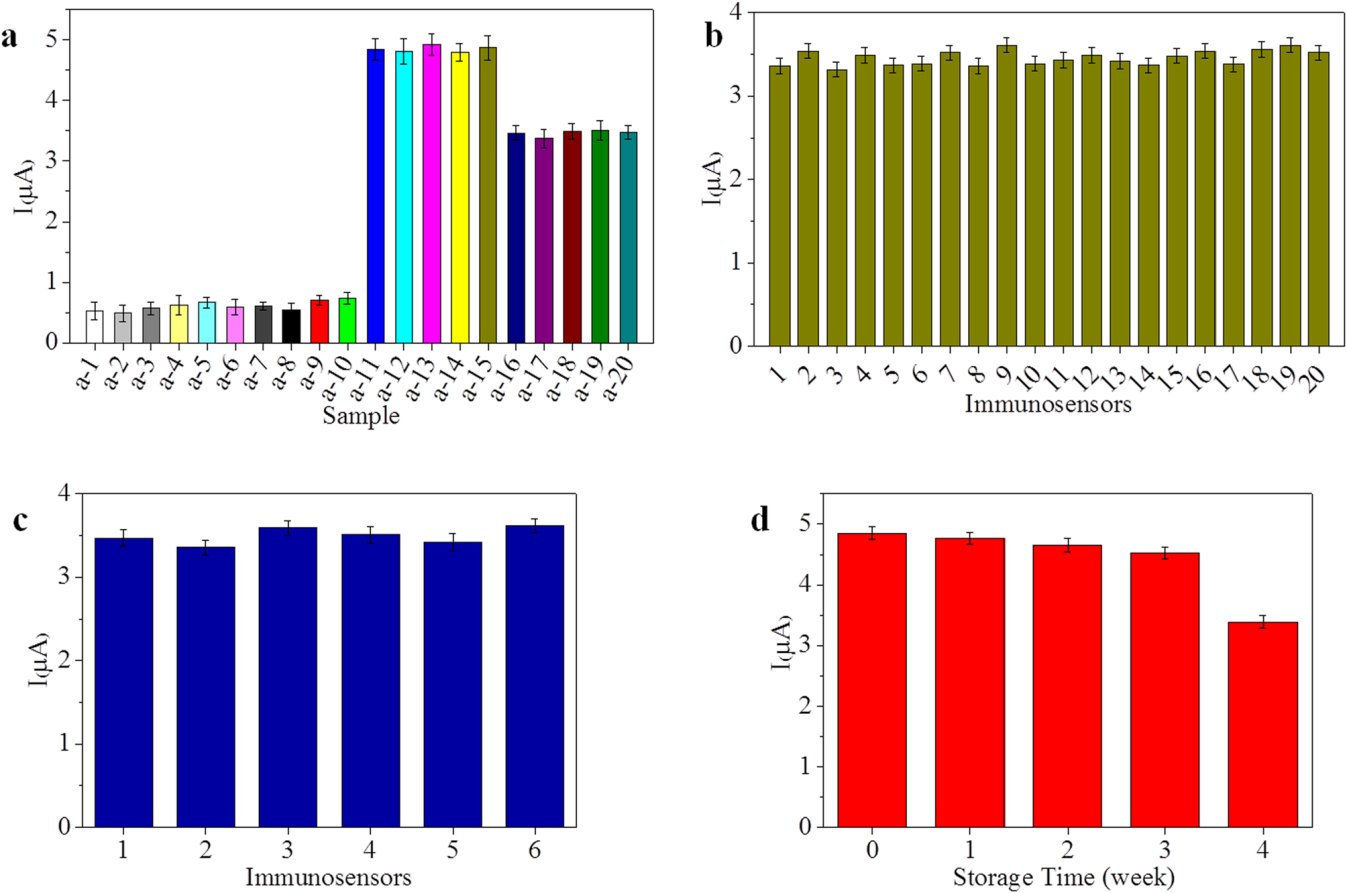
(**a**) Selectivity of the immunosensor: (a-1) ddH_2_0, (a-2) AAV (10^6.37^ EID_50_/0.1 mL), (a-3) IBV (10^7.02^ EID_50_/0.1 mL), (a-4) ILTV (10^5.84^ EID_50_/0.1 mL), (a-5) AIV H7 (10^6.45^ EID_50_/0.1 mL), (a-6) ARV (10^6.51^ EID_50_/0.1 mL), (a-7) IBD (10^7.34^ EID_50_/0.1 mL), (a-8) glucose (1.0 µg/mL), (a-9) vitamin C (1.0 µg/mL), (a-10) BSA (1.0 µg/mL), (a-11) NDV (10^5.13^ EID_50_/0.1 mL), (a-12) NDV (10^5.13^ EID_50_/0.1 mL) + AAV (10^6.37^ EID_50_/0.1 mL), (a-13) NDV (10^5.13^ EID_50_/0.1 mL) + IBV (10^7.02^ EID_50_/0.1 mL), (a-14) NDV (10^5.13^ EID_50_/0.1 mL) + AIV H7 (10^6.45^ EID_50_/0.1 mL), (a-15) NDV (10^5.13^ EID_50_/0.1 mL) + ARV (10^6.51^ EID_50_/0.1 mL), (a-16) NDV (10^3.13^ EID_50_/0.1 mL), (a-17) NDV (10^3.13^ EID_50_/0.1 mL) + ILTV (10^5.84^ EID_50_/0.1 mL), (a-18) NDV (10^3.13^ EID_50_/0.1 mL) + IBD (10^7.34^ EID_50_/0.1 mL), (a-19) NDV (10^3.13^ EID_50_/0.1 mL) + vitamin C (1.0 µg/mL), and (a-20) NDV (10^3.13^ EID_50_/0.1 mL) + BSA (1.0 µg/mL); (**b**) repeatability, (**c**) reproducibility, and (**d**) storage stability of the immunosensor.

Selectivity is a significant parameter for an immunosensor. Therefore, to determine the selectivity of the fabricated immunosensor, some possible interferents, including aviadenovirus group I (AAV, 10^6.37^ EID_50_/0.1 mL), infectious bronchitis virus (IBV, 10^7.02^ EID_50_/0.1 mL), infectious laryngotracheitis virus (ILTV, 10^5.84^ EID_50_/0.1 mL), avian influenza virus subtype H7 (AIV H7, 10^6.45^ EID_50_/0.1 mL), avian reovirus (ARV, 10^6.51^ EID_50_/0.1 mL), infectious bursal disease (IBD, 10^7.34^ EID_50_/0.1 mL), glucose (1.0 µg/mL), vitamin C (1.0 µg/mL) and BSA (1.0 µg/mL), were investigated. The results are depicted in Fig. 6a. When the fabricated immunosensor was exposed to possible interferents (Fig. 6a (samples a-2 ~ a-10): AAV, IBV, ILTV, AIV H7, ARV, IBD, glucose, vitamin C, and BSA), the detection currents were as low as that for the negative control (Fig. 6a (sample a-1): ddH_2_0). The immunosensor exhibited a higher signal when incubated with a sample including NDV (Fig. 6a (samples a-11, a-16)) than when incubated with samples containing the possible interferents (Fig. 6a (samples a-2 ~ a-10)). Additionally, the responses of the fabricated immunosensor to 10^5.13^ and 10^3.13^ EID_50_/0.1 mL NDV solutions containing other interfering substances were measured (Fig. 6a (samples a-12 ~ a-15, a-17 ~ a-20)), and the current variation due to the interfering substances was less than 5% of that obtained without interferences. The results show that the developed immunosensor had good selectivity for NDV.

Under the optimal experimental conditions, equivalently prepared immunosensors were used to detect 10^3.13^ EID_50_ NDV 20 times to evaluate the repeatability of the developed immunosensor, and the results are shown in Fig. 6b. The relative standard deviation was 2.58%, demonstrating the good repeatability of the immunosensor. The reproducibility of the immunosensor was evaluated by preparing six different batches of the immunosensor independently. A series of six different batches of the immunosensor were prepared for the detection of 10^3.13^ EID_50_ NDV, and the results are shown in Fig. 6c. The relative standard deviation was found to be 2.84%, showing the excellent reproducibility.

Long-term storage stability tests show the robustness of an immunosensor. The current responses of the developed immunosensor were periodically checked to evaluate its stability. The immunosensor was stored in PBS (pH = 7.4) at 4 °C when it was not in use. Every week, electrochemical measurements were performed with the developed immunosensor, and the average value was calculated based on five assays. The results shown in Fig. 6d indicated that the immunosensor response current decreased by only 4.1% after 2 weeks. After four weeks, the immunosensor current response decreased by 9.5% relative to its initial current, which indicated that the immunosensor had acceptable storage stability.

### Application of the proposed immunosensor for the detection of NDV

Oral and cloacal swab samples, which were gently collected from fowls at different live bird markets in Guangxi Province, were used as clinical samples. A viral transport medium composed of 0.05 mmol/L PBS containing 10 mg/mL gentamycin, 10 mg/mL kanamycin, 10 mg/mL streptomycin, 5% (v/v) foetal bovine serum and 10,000 units/mL penicillin was used to prepare the clinical samples, and the clinical samples were placed in an ice box.

With the permission of the owners of the live bird markets, a total of 120 clinical samples were collected from chickens, the samples were assayed using the proposed immunosensor, and seven NDV-positive samples were detected. Virus isolation^3^ was employed to confirm the test results. The positive results detected by the developed immunosensor were in agreement with the results of virus isolation, and the results are summarized in Table 1b,c. To test the recovery by the proposed immunosensor, NDV standards were added to the clinical samples that had been confirmed as positive. The results (Table 1d) showed that the fabricated immunosensor had acceptable recovery (96.28 ~ 104.49). Considering the acceptable recovery in real samples, the immunosensor was found to be practical for sample detection.

## Materials and methods

### Reagents and materials

MAb/NDV and PAb/NDV were purchased from Abcam (Cambridge, UK). Copper sulfate (CuSO_4_), hydrochloroauric acid (HAuCl_4_), graphite powder (< 45 mm), KMnO_4_, NaNO_3_ and H_2_SO_4_ were supplied by the Guoyao Group Chemical Reagents Co., Ltd., Shanghai. Bovine serum albumin (BSA) was purchased from Sigma (USA). All chemicals used were of analytical reagent grade. Double-distilled deionized water was used in all experiments. In addition, 10 mmol/L PBS (pH = 7.4) was prepared by mixing stock solutions of 10 mmol/L NaH_2_PO_4_ and 10 mmol/L Na_2_HPO_4_.

### Instruments

SEM was performed on a HITACHI UHR FE-SEM SU8000 Series (SU8020) instrument. FT-IR spectra were collected on a Nicolet IS10 instrument. XPS analysis was performed on an X-ray photoelectron spectrometer (ESCALAB 250Xi, Thermo Scientific). A CHI660D electrochemical workstation (Beijing CH Instruments, Beijing, China) with a standard three-electrode cell (a working electrode, an SCE as the reference electrode and a platinum wire as the auxiliary electrode) was employed to study the electrochemical characteristics. Electrochemical detection was performed at room temperature (25 ± 0.5 °C).

### Gra synthesis

A modified Hummers method was used to prepare Gra oxide^34^. In short, NaNO_3_ (2.5 g) and graphite powder (1.0 g) were added to concentrated H_2_SO_4_ (100 mL) and stirred for 2 h. KMnO_4_ (5 g) was slowly added to the mixture under continuous stirring, and the mixture was then cooled with ice. Next, the mixture was stirred at 35 °C for 24 h. Double-distilled deionized water (100 mL) was slowly added to the reacted slurry, which was then stirred at 80 °C for another 3 h. Next, more double-distilled deionized water (300 mL) was added to the reacted slurry. Then, 6 mL of H_2_O_2_ (30%) was added (bubbles appeared, and the slurry immediately turned bright yellow). The resulting solution was continuously stirred for 3 h and then precipitated for 24 h at room temperature. The supernatant was subsequently decanted. The resulting yellow slurry was washed with 0.5 mol/L HCl (500 mL) and centrifuged. The solution was washed with double-distilled deionized water and centrifuged until the pH of the solution was neutral (pH = 7.0). Gra oxide was obtained after the solution was ultrasonicated for 2 h. To obtain Gra, Gra oxide was reduced at 95 °C for 3 h using NaBH_4_ as a reducing agent.

### Preparation of the Chi-Gra nanocomposite

Chi*-*Gra was prepared according to a previously reported method^23^. Briefly, Chi powder was dissolved in a 1.0% (v/v) acetic acid solution under stirring for 0.5 h at room temperature until it was completely dispersed. The Chi solution (0.5 wt.%) was thus prepared. Then, Gra (10 mg) was added to the Chi solution (10 mL), ultrasonicated for 1 h, and stirred for 24 h at 25 °C. Finally, the Chi-Gra nanocomposite was obtained.

### Preparation of the AuNP-Chi-Gra nanocomposite

The AuNP-Chi-Gra nanocomposite was prepared as previously described^23,35^. Furthermore, 0.5 mL of HAuCl_4_ (1 mM) was added to Chi-Gra (5 mL) under stirring at 25 °C for 4 h. Then, the solution was incubated at 80 °C for 1 h with vigorous stirring. Au^3+^ was subsequently reduced to AuNPs by Chi at 80 °C. Finally, the AuNP-Chi-Gra nanocomposite was obtained.

### Preparation of the Cu(I)/Cu(II)-Chi-Gra nanocomposite

The Cu(I)/Cu(II)-Chi-Gra nanocomposite was prepared according to the method used to prepare the AuNP-Chi-Gra nanocomposite with certain modifications. CuSO_4_·5H_2_O was used as the source of copper. First, 10 mg of CuSO_4_·5H_2_O was added to 5 mL of the Chi-Gra nanocomposite under continuous stirring at 25 °C for 8 h. Then, the mixture was incubated at 95 °C for 4 h under continuous stirring. Finally, the Cu(I)/Cu(II)-Chi-Gra nanocomposite was obtained.

### Preparation of PAb/NDV-Cu(I)/Cu(II)-Chi-Gra nanocomposite bioconjugates

First, 5 mL of the Cu(I)/Cu(II)-Chi-Gra nanocomposite obtained from the above preparation method was centrifuged (12,000 rpm, 10 min), the supernatant was discarded, and the residue was washed with double-distilled deionized water three times to remove the excess Chi, Cu^2+^ and SO_4_^2−^ that did not combine with Gra. Then, 5.0 mL of a PBS buffer (pH = 7.4) was added to the residue to disperse the Cu(I)/Cu(II)-Chi-Gra nanocomposite, and the mixture was sonicated for 10 min to obtain a homogeneous suspension. Next, 1 mL of PAb/NDV (10 µg/mL) was added to the homogeneous suspension, and the mixture was vigorously stirred for 5 min at 4 °C. Then, 1 mL of 1% glutaraldehyde was slowly added to the solution under continuous stirring. The solution was subsequently incubated at 4 °C for 8 h. The reaction mixture was washed with PBS (pH = 7.4) and centrifuged (12,000 rpm, 10 min) three times. The supernatant was discarded, the resulting mixture was dispersed in PBS (5.0 mL, pH = 7.4), and 1 mL of a 2.0% (w/v) BSA solution was added to the suspension, which was then incubated at 4 °C for 8 h. The obtained PAb/NDV-Cu(I)/Cu(II)-Chi-Gra nanocomposite was stored at 4 °C for further use.

### Fabrication of the electrochemical immunosensor

First, 0.05 mm alumina was used to polish a GCE (Ø = 3 mm) until it had a mirror-like surface. Then, the GCE was rinsed with double-distilled deionized water and ultrasonicated in baths of double-distilled deionized water, ethyl alcohol, and double-distilled deionized water to remove any physically adsorbed substances. Next, the GCE was placed in H_2_SO_4_ (0.05 M) and chemically cleaned until the background signal stabilized. Finally, the GCE was thoroughly rinsed with double-distilled deionized water and dried with nitrogen gas to obtain a clean GCE.

**Figure 7:**
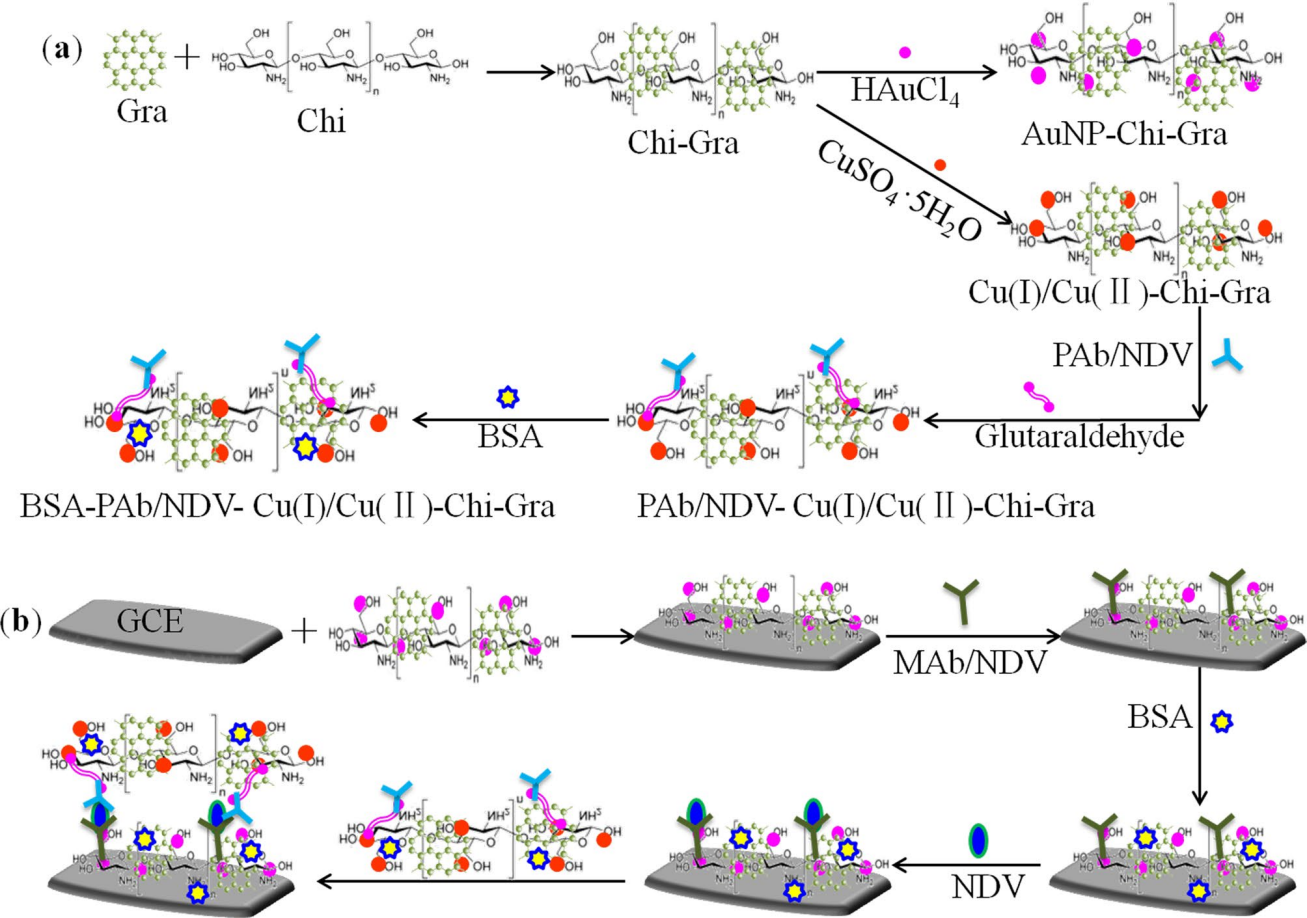
Preparation procedures of AuNP-Chi-Gra, Cu(I)/Cu(II)-Chi-Gra (**a**) and the immunosensor (**b**).

Figure 7 shows the procedures used to construct the immunosensor. The process was as follows: the AuNP-Chi-Gra (8 μL) nanocomposite was dropped onto the clean GCE surface, dried at 4 °C overnight to obtain the modified electrode (AuNP-Chi-Gra-GCE), washed with double-distilled deionized water, immersed in a 1 µg/mL (200 µL) MAb/NDV PBS solution (pH = 7.4) and incubated at 4 °C for 8 h. The resulting electrode (MAb/NDV-AuNP-Chi-Gra-GCE) was immersed in a 1.0% (w/w) BSA solution for 1 h at 37 °C to block the remaining active sites. The final modified electrode was stored at 4 °C when not in use.

### Electrochemical immunosensor detection

A well-known sandwich immunoassay was used to detect NDV. First, the MAb/NDV-AuNP-Chi-Gra-GCE immunosensor was incubated with 15 μL of the sample for 30 min and then washed with a PBS buffer (pH = 7.4) to remove non-specifically adsorbed conjugates. Next, the modified electrode was incubated with 200 μL of the PAb/NDV-Cu(I)/Cu(II)-Chi-Gra nanocomposite for 40 min and washed with a PBS buffer (pH = 7.4). Finally, the resulting electrode was placed in a 0.01 mol/L PBS (pH = 7.4) KCl solution, and DPV experiments were performed (− 0.3 to 0.4 V, 50 mV/s) to detect NDV.

### Ethics statement

The authors confirm that relevant guidelines were followed for the care and use of animals. This work was approved and conducted by the Animal Ethics Committee of the Guangxi Veterinary Research Institute, which supervises all live bird markets in Guangxi Province. Oral and cloacal swab samples, which were gently collected from fowls at different live bird markets in Guangxi Province, were used as clinical samples. Before sampling, the fowls were not anaesthetized, and after sampling, they were returned to their cages and observed for 30 min.

## Conclusions

In summary, AuNP-Chi-Gra was used as a platform, and PAb/NDV-Cu(I)/Cu(II)-Chi-Gra was used as a label for signal amplification in this work. Based on the well-known sandwich immunoreaction, a novel electrochemical immunosensor was developed for the quantitative detection of NDV. It exhibited a linear response over a wide range (10^0.13^ to 10^5.13^ EID_50_/mL), had a low detection limit (10^0.68^ EID_50_/0.1 mL), and was more sensitive than an immunosensor with PAb/NDV-Cu(I)/Cu(II)-Chi as the signal label (the limit of detection for NDV was 10^2.09^ EID_50_/0.1 mL). This newly designed immunosensor might have widespread application potential because it had acceptable reproducibility, selectivity and stability; could be obtained by a facile fabrication procedure; and was ultrasensitive for the detection of NDV.
